# Screening, brief intervention, and referral to treatment for risky stimulant use in a Skid Row community health center

**DOI:** 10.1186/1940-0640-7-S1-A11

**Published:** 2012-10-09

**Authors:** Lillian Gelberg, Ronald M Andersen, Lisa Arangua, Henry Teaford, Niree Hindoyan, Sareen Malikian, Jose C Muniz Castro, Hugo Yepez, Mani Vahidi

**Affiliations:** 1 University of California/Los Angeles Center for Health Policy Research, Los Angeles, CA, USA; 2 David Geffen School of Medicine, University of California/Los Angeles, CA, USA

## 

The University of California at Los Angeles Quit Using Drugs Intervention Trial (QUIT) aims to conduct a randomized controlled trial of a primary-care based very brief intervention protocol for reducing risky stimulant use and drug-related harm in low-income, racially diverse primary care patients attending safety-net clinics in the east central Skid Row area of Los Angeles. The QUIT trial emphasizes screening, very brief clinician advice (2-3 minutes), and two telephone drug-use health education sessions versus usual-care in the control group (240 patients per condition). Between February 18 and April 28, 2011, pre-visit screening of adults in the waiting room was conducted using a touch-screen Tablet PC. “At risk” drug use was defined as casual, frequent, or binge use without the physiological or psychological manifestations of dependence (a score of 4 to 26 on the World Health Organization’s Alcohol, Smoking, and Substance Involvement Screening Test (ASSIST). A total of 920 adult patients were approached: 89% were 40+ years old; 68% were male; 62% were black, 21% were Latino, and 17% were white. Of patients approached, 706 were excluded due to pregnancy, because it was a nonprimary care visit, or because they refused to participate. Among the 214 who completed the ASSIST, substance use scores were none or low risk in 11% of participants, moderate risk in 42%, and dependence-level in 47%. The number of participants in each score range by substance were, respectively, tobacco 55, 101, and 58; alcohol 62, 98, and 54; cannabis 94, 77, and 43; cocaine 89, 74, and 51; methamphetamine/amphetamine type stimulants 145, 45, and 23; inhalants 185, 20, and 9; sedatives 143, 45, and 26; hallucinogens 174, 30, and 10; and opioids 130, 54, and 30. Participants who were older than 50 years were more likely to use tobacco, alcohol, cannabis, and cocaine; younger patients were more likely to use amphetamines, inhalants, sedatives, hallucinogens, and opioids. Twenty-seven patients (3% of those approached) met study criteria of past three-month risky stimulant use. Seventy percent were homeless, and 30% were marginally housed. In Skid Row, only 3% of patients qualified for risky stimulant use intervention.

